# The Influence of SNS on Policy Support to Mitigate Public Health Crises: The Mediating Role of General and Personal Risk Perceptions

**DOI:** 10.3390/ijerph191710933

**Published:** 2022-09-01

**Authors:** Soohee Kim

**Affiliations:** Department of Communication Contents, Dongduk Women’s University, Seoul 02748, Korea; soohee@dongduk.ac.kr

**Keywords:** SNS dependency, general risk perception, personal risk perception, policy support, environmental health risks

## Abstract

Recent evidence suggests that social networking sites (SNS) motivate people, in the form of civic engagement, in times of crisis. Yet, there is a lack of empirical investigations that help to understand how SNS use increases civic judgment or participatory behavior. In this study, we examine how the use of SNS in a public health crisis is related to policy support for effective mitigation of risk, particularly focusing on the role of two distinct types of risk perceptions—general and personal risk perceptions. Using an online survey conducted on the issue of fine dust pollution in South Korea (N = 510), this study found that reliance on SNS for learning (i.e., SNS learning dependency) is positively associated with general risk perception, which, in turn, promotes policy support. Moreover, the results revealed a serial mediation process in which SNS learning dependency increases general risk perception, which ultimately increases personal risk perception and policy support. Overall, this study suggests that SNS has the potential to facilitate public engagement in a crisis and that individual motivation to engage with a crisis is not just a function of individual or societal-level risk perception but can be a simultaneous function of different levels of risk perceptions.

## 1. Introduction

Social networking sites (SNS) have become prominent communication platforms that people use in the context of risks. By sharing relevant news and information [1,2], expressing emotions [3,4], or posting relevant pictures or videos [5], people have increasingly used SNS as a tool for communicating about crises. In particular, it has been linked to some desirable civic consequences contributing to overcoming problems associated with the risk. For example, SNS use has been found to boost people’s interest in social problems [6], participation in collective action against the risk [7], or support for measures relevant in addressing the problem [8]. Indeed, even though there is still some debate about whether SNS can be a means of civic and political engagement [9,10], it is now generally believed that such platforms possess the potential to motivate people in forms of civic engagement and participation in crisis contexts [11].

While considerable research has examined the impact of SNS on people’s engagement with risk issues, notably little research has been conducted to investigate the mechanisms underlying the impact of SNS on judgment and behavior. In fact, studies on SNS have primarily focused on identifying the causes or consequences of SNS use [5] or analyzed the contents of crisis-related discourse or information on SNS [12], rather than examining the cognitive or affective mechanisms underlying its impact. As such, the processes that describe how and why SNS can be linked to public engagement with risks are largely unknown.

The present study aims to bridge this gap by focusing on the role of risk perceptions in the process. Defined as individuals’ subjective judgments of the likelihood that negative events may occur [13], risk perceptions have been identified as crucial variables impacting people’s judgment and decision-making in the context of risks [14]. In addition, the literature on media effects has consistently suggested that the perceptions of risk are largely influenced by the way individuals use media [15,16] and that new media, such as SNS, exerts a powerful influence on judgments about risks [17,18]. In particular, the literature on risk perception has documented that the role of risk perceptions in media effects can be differentially understood, depending on the perceived target of the risk (i.e., who is at risk). That is, the influence of media on people’s judgment and behavior may vary depending on how media influences the perception of who is vulnerable to the risk [15,19]. While this points to the possibility that SNS use is distinctively associated with perceptions of different risk targets (e.g., social vs. personal), thus influencing risk-related judgment or behavior, few studies have empirically tested the potentially distinctive role of perceived targets of the risk in examining the impact of SNS.

Therefore, the goal of the present study is twofold. First, we provide an empirical examination of how SNS use is associated with people’s engagement and participation in crisis contexts. Second, we investigate the role of risk perception as a possible cognitive mechanism, particularly paying attention to the potentially distinctive role of two different levels of risk perceptions (i.e., general and personal risk perceptions) in the process. In doing so, the present study utilizes the concept of SNS dependency to better understand the multidimensional impact of SNS on individuals’ judgment and behavior [20,21]. To the best of our knowledge, this study is the first to explore the potentially distinctive roles of general and personal risk perceptions in the impact of SNS dependency.

We address these questions in the context of South Korea’s fine dust pollution. The average ultrafine dust concentration in South Korea is the worst among the OECD countries [22]. South Koreans consider fine dust as the most pressing environmental problem that must be solved. Reflecting this public concern about the fine dust problem, South Korea’s National Assembly has officially declared fine dust pollution as a “social disaster”. We believe the issue of fine dust air pollution in South Korea provides an interesting avenue to observe the interactions among SNS use, risk perceptions, and policy preferences. The examination of how SNS use impacts public engagement and support for policies to mitigate the risk will promote our understanding of the mechanism underlying the social impact of SNS.

## 2. Theoretical Background

### 2.1. SNS as a Platform for Communication in Public Health Crises

In times of public crises, the media plays a primary role in shaping people’s beliefs about or judgment of the crises [23,24]. As people often have limited knowledge or expertise about public or social issues—especially the ones from the health or environmental domains—the media exerts a powerful influence on people’s responses to these problems [25,26]. In particular, since becoming a dominant form of communication in today’s media environment, SNS has been increasingly used as a major communication tool in the midst of crises. Research evidence presents that it now serves as a major platform to link individuals to critical information in a real-time basis [3], and people increasingly turn to SNS to seek information on how to deal with the crisis in question, as well as express and share crisis-related emotions [27].

Extant literature suggests that the powerful impact of SNS in crisis situations is largely explained by the unique nature of SNS communication. First, being different from mass media, in which information is primarily received from official sources or institutions, SNS allows individuals to learn from friends or others close to them in their network. Accordingly, this information from their own network is considered more personally relevant [28], increasing their willingness to act on the problem.

Additionally, news and information on SNS does not only exist in the form of factual information but also personal experiences and stories. The postings, comments, or video uploads of friends or close acquaintances that deliver vivid stories related to a given issue makes the information more compelling and invokes stronger emotional responses than objective or statistical information, thus more effectively affecting people’s beliefs and attitudes [29]. In fact, the literature suggests that, when information takes the form of narratives (i.e., stories), its persuasive effect increases; as a recipient is transported by the “lived experiences” of others, it becomes harder for them to counterargue, thereby being more likely to be persuaded by the message [30] p. 180. Furthermore, compared to traditional media, news or information on SNS is generally perceived to be more trustworthy, enhancing its persuasive power. While traditional news media are considered to deliver news from public sectors or certain organizations that may have specific goals and purposes, information on SNS, coming from personal networks, is considered to be more honest and closely reflects the reality of the situation. The literature also suggests that SNS can introduce social pressure that motivates people to accept others’ attitudes and behaviors [31]. For example, when exposed to a friend’s or other network member’s post presenting how they feel about a certain problem (e.g., how serious the problem is) and react to it (e.g., participation in rally, writing a petition), the trust and obligation embedded in the relationship incites social pressure in the individual to take part in such significant actions.

Empirical studies on risk communication indicate that SNS use is, indeed, linked to a range of important cognitive, emotional, and behavioral consequences in the context of crises. For instance, SNS was shown to promote people’s knowledge and information about the risk [5], facilitate the sharing of emotions [32], which boosts empathic concerns for other people [3], and lead to various types of risk-preventive behaviors [4]. In essence, SNS—as a major communication platform in which people share information and emotion in times of public crisis—may exert powerful influence on people’s beliefs and perceptions about the risk, which, in turn, shapes important judgments about the risk, such as how to cope with the situation.

### 2.2. SNS Dependency

Many studies have examined the impact of SNS by focusing on the time spent on SNS or frequency of use of SNS [33]. However, research has begun to emphasize the importance of examining the multifaceted aspects of SNS use: for example, focusing on SNS connectedness [34], SNS intensity [35], or SNS dependency [20]. In particular, research has increasingly demonstrated that individuals’ reliance on SNS to achieve their everyday goals (i.e., SNS dependency) is critically associated with their attitudes and behavior regarding social problems or issues. Defined as “a relationship in which the capacity of individuals to attain their goals is contingent upon the capacities of SNS to create, gather, process, and disseminate information” [20] p. 12, the concept of SNS dependency originally evolved from the theory of media system dependency (MSD) [36]. MSD posits that individuals carefully weigh the usefulness of media for fulfilling their goals. Specifically, the theory proposes that media effects are more pronounced when the individual is highly dependent on the media to achieve one’s specific goals [37]. According to the theory, media dependency comprises different subsets of goals: orientation (personal and social action), understanding (personal and social), and play (solitary and interaction) [37]. Thus, as individuals have different goals for using media, it becomes critical to consider the specific goals, for which individuals rely on media, to better understand its impact.

Based on the literature, we examine two types of SNS dependency—SNS learning dependency and SNS entertainment dependency—as critical aspects of SNS dependency. First, SNS learning dependency relates to two main goals of media dependency: action and understanding. These goals concern how people use media resources for obtaining guides to specific behaviors of their own (orientation) and for understanding events, cultures, and people around them (understanding). In addition, SNS entertainment dependency concerns the goal of play as it relates to how individuals use SNS to entertain themselves. We believe this concept of SNS dependency is particularly useful in understanding the impact of SNS in crisis contexts because MSD proposes that higher media centrality and more problematic social situations work together to increase MSD relations [36]. Thus, when a problem is more problematic, for example, because people are uncertain about the causes and consequences of the problem or how to approach and solve the issue, individuals’ reliance on SNS will be more likely to increase, resulting in greater media reliance and its impact. Therefore, the present study utilizes the conceptualization of SNS dependency (learning, entertainment) to better comprehend the impact of SNS on perceptions and judgments in times of crises.

### 2.3. SNS Dependency and Risk Perceptions

While SNS may influence various aspects of perceptions and beliefs about risks, one factor likely to play a crucial role in the public response to crises is the perception of risk, i.e., the extent to which people perceive a given issue as risky or threatening [13]. Considerable work has demonstrated that risk perceptions serve as antecedents to various ranges of risk-related judgments or behaviors, such as the intention to support policies to address the risk [8], participate in risk-preventive behavior [38], and join collective actions [7].

In particular, the literature on risk perception has emphasized that its effects can significantly vary depending on the targets of risk [19]. This line of research indicates that, depending on the presumed target of risk, people form different judgments about how risky or threatening a problem will be. These studies, in particular, suggest that people nearly always tend to judge personal risk to be lower than a general one [15]. Often conceptualized as optimistic bias [39], this tendency leads people to underestimate the risk to themselves compared to others. Studies on media and risk perception have shown that this tendency is clearly shown in the presumed impact of media on individuals. For instance, it has been theorized that mass media messages are less likely to influence individuals’ perceptions of risk for themselves; instead, it induces the perception that the risk poses a threat to generalized others (Impersonal Impact Theory) [40]. However, other studies have proposed a different possibility. For example, considering the potentially distinct effects that different types of media might have on risk perceptions, research on the differential impact hypothesis has proposed that different types of media, such as informative and entertainment media, may have different impacts on risk perceptions. Specifically, entertainment media can lead to greater personal risk perceptions than news media because entertainment media describe events vividly, which makes the risk more salient and relevant to the self [25]. These theoretical explanations point out that different types of media—or differential uses of media—might have distinct influences on perceptions about the targets of risk.

Existing research has explored the potentially distinct impact of media on perceptions about the presumed target of risk. For example, Han et al. [41] examined the differences in personal and general risk perceptions to a H1NI flu health risk at four different levels (personal, group, societal, global), focusing on how different types of media, such as traditional media and the Internet, SNS, and interpersonal communication exert differential influences on those perceptions. Their findings showed that SNS is a significant predictor for risk perception, particularly for the general rather than the personal level, in both contexts. Specifically, SNS exposure increased group and societal-level risk perceptions in the United States and societal and global-level risk perceptions in China. On the contrary, SNS exposure did not predict personal level risk perceptions in either of the two countries. While their study focused on how different types of communication channels may influence risk perceptions distinctively, their findings suggest that SNS can, indeed, be more successful in inducing perceptions of risk for people, in general, than for the self.

Based on the theories and research evidence, the present study predicts that SNS use will be associated with the perception of general risk—the perception that the general public may be harmed by the fine dust problem. In particular, the present study expects that SNS learning dependency is positively related to the perception that the risk issue can be a threat to people in general. As discussed earlier, when individuals rely on SNS for learning, they have ample opportunities to obtain information about critical social issues and to learn other people’s thoughts, emotions, and responses to those issues. Thus, while using SNS to learn more about society and people, individuals are likely to increase their understanding of how a given issue/risk affects other people’s lives, which may increase the perception that the risk can pose a threat to the general population. Thus, we raise the following hypothesis.

 **H1.** *SNS learning dependency is positively related to the perception of general risk.*

On the contrary, we expect that SNS learning dependency is less likely to be associated with the perception that the risk poses a serious threat to oneself. As the optimistic bias literature suggests, people might want to believe that they are less susceptible to the danger [39]. Thus, they may consider the risk described in the media to be less relevant to themselves. However, there is a possibility that SNS learning dependency might induce personal risk perception. Individuals often interact with other people on SNS while observing others’ thoughts and behaviors, and this process may amplify the personal relevance of social issues and messages. In line with this reasoning, some studies found the positive influence of SNS use on personal risk perception. For instance, Wu and Li [18] examined how mass media use and SNS involvement (i.e., the extent to which people use online platforms to communicate with others sharing a relationship) influence risk perceptions and precautionary behavior in the context of environmental risk. They found that SNS involvement affects personal risk perception which, then, impacts precautionary behavior. Similarly, Lee et al. [42] demonstrated that receiving information through SNS was positively related to all levels of risk perceptions—personal, group, and global risk perceptions. However, considering that other studies did not find the association of SNS use with personal level risk perceptions [41], we examine the relationship between SNS dependency for learning and the perception of personal risk as a research question.

 **RQ1:** Is SNS learning dependency related to the perception of personal risk?

It is also complicated to predict the influence of SNS entertainment dependency on general and personal risk perceptions. On the one hand, it is possible that the use of SNS for entertainment is positively related to perceptions of generalized risk for others and for self. Regardless of the actual content individuals use on SNS, people might experience closeness and intimacy with other people while closely interacting with them, thus developing more empathic perceptions and concern for people in general. Previous work also showed that the use of SNS for entertainment increased thoughts about other people and participatory behaviors [43]. On the other hand, reliance on SNS mainly for entertainment may not relate to the perception that a risk might affect others or self. Posting photos, updating status, or playing games on SNS, as purely entertainment-seeking activities, are less likely to be associated with thoughts or perceptions about risk issues. In fact, research has consistently documented that these entertainment-oriented activities on SNS are more likely linked to personal interests and activities rather than public interests and civic engagement [26]. This suggests that using SNS for play or entertainment may undermine individuals’ sense of reality and their interests and involvement with important real-world issues, thus leading to decreased thoughts and perceptions about social problems.

Therefore, due to the different possibilities regarding the impact of SNS entertainment dependency on personal and general risk perceptions, we pose this as a research question.

 **RQ2:** Is SNS entertainment dependency related to general or personal risk perceptions?

### 2.4. SNS Dependency and Policy Support

Beyond the impact of SNS use on risk perceptions, SNS use may directly relate to judgment or behavioral intentions to engage with social problems. An abundance of research documents how SNS may impact risk-related judgment or behavior. For example, SNS facilitates interest in other people’s lives and participation in local or social problems [44] because SNS effectively increase conversations and interactions with others. People can freely interact with others with low cost of communication, which, in turn, increases their intention to engage with social problems. The various functions and features of SNS have also been shown to contribute to the development of civic concerns. For example, research shows that the use of hyperlinks—widely used in social media settings—leads individuals to access a number of resources that stimulate their interests in various ranges of social issues [45].

Empirical studies have found that SNS use is, indeed, linked to participation in community or public crisis issues. For instance, Lavertu et al. [46] found that SNS promote prosocial behavior, such as the likelihood of making a donation, particularly when online audiences become more salient. They explain that SNS use can increase such behavior due to heightened public self-awareness and extrinsic motivations. Kim et al. [20] and their study also showed that increased SNS dependency is positively linked to community activity engagement. They explain that SNS has a “pull effect” as a facilitator of such engagement, as it allows people to discuss local and social issues more easily while providing venues for local storytelling. Similarly, Kim and Jung [47] showed that a higher level of SNS dependency was associated with more active engagement with interactive activities such as sharing stories about politics with others. Taken together, we expect that individuals’ reliance on SNS to understand the meaning of events and issues around them can be positively linked to engagement in actions to address social problems, including the support for policies to mitigate the problems. On the contrary, reliance on SNS for entertainment is likely not associated with engagement with social problems since entertainment-oriented activities would rarely increase conversations about community/social issues or access to information that motivate their interests in public issues. Previous work also demonstrated that SNS use for entertainment was not or negatively related to individuals’ civic participation [48]. Thus, the following hypothesis is posed.

 **H2.** *SNS learning dependency (not SNS entertainment dependency) is positively related to the support for policies to mitigate risks.*

### 2.5. Risk Perceptions and Policy Support

It has been widely established that risk perception is a primary variable predicting public policy support [13]. Various theories, such as the protection motivation theory [49] and health belief model [50], suggest that individuals’ beliefs of being vulnerable to a risk emerge as a strong motivation for people to engage in behaviors to mitigate the risk. Indeed, research evidence has consistently demonstrated that risk perception is the critical factor for changes in judgment or behaviors, such as engaging in preventive behaviors [38] and supporting policies to address problems associated with the risk [51]. In general, these studies suggest that risk perceptions—regardless of general and personal risk perceptions—lead people to engage in behaviors that can help address the risk [19]. Thus, we pose the following hypotheses.

 **H3.** *The perception of general risk is positively related to the support for policies to mitigate risks.*

 **H4.** *The perception of personal risk is positively related to the support for policies to mitigate risks.*

The literature on social psychology and communication has long documented that an individual’s perception of people, in general, being threatened by a particular risk can induce the perception that he or she is also threatened by it. For instance, social norm theories propose that people form their attitudes and judgments about social issues by looking to others due to informational and/or normative influences [52]. According to this theory, individuals’ judgment or attitudes are affected by what they perceive other people think because of “informational influence”—where individuals yearn to be correct about their attitudes and judgment—and/or “normative influence”—where people want to belong to or be accepted by other people. Thus, in the context of risk communication on SNS, general risk perception can be positively linked to personal risk perception: while reading posts and comments of other people and checking real-time information updated on SNS, people may learn how the issue impacts everyone’s lives, and this understanding, in turn, may lead to a recognition that the issue can affect themselves as well. Indeed, empirical studies found a strong correlation between the perceptions of risk to others and oneself [53]. Lee et al. [42] also demonstrated that people who frequently use SNS tend to relate the crisis to themselves as well as their communities, becoming motivated to engage in information seeking and preventive behaviors. Thus, we raise the following hypothesis.

 **H5.** *The perception of general risk is positively related to the perception of personal risk.*

### 2.6. Mediating Role of Risk Perceptions

Based on the discussion above, we expect a mediation process where the influence of SNS learning dependency is affected by risk perceptions. Specifically, to the extent that SNS learning dependency is linked to general risk perceptions, and these perceptions are predictive of policy support, the perception of general risk may mediate the impact of SNS learning dependency. Previous studies also showed that perceptions of risk mediate the impact of media on public judgment and behavior in various types of risk contexts [54]. Therefore, the current study predicts that the influence of SNS learning dependency on the intention to support policies is mediated through the perception that people, in general, are threatened by the risk.

 **H6.** *SNS learning dependency will influence the support for polices to mitigate a risk through the perception of general risk.*

In addition, based on this reasoning and the insights gained from the previous literature, we expect a serial mediation model where SNS learning dependency affects the general risk perception which, in turn, influences the personal risk perception and, then, policy support. Thus, we pose the following hypothesis.

 **H7.** *SNS learning dependency will influence the support for polices to mitigate a risk through the general and personal risk perceptions, serially.*

**Figure 1:**
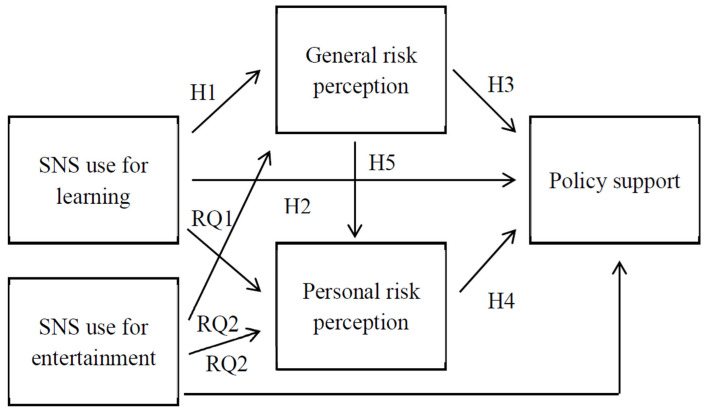
Hypothesized model of the study.

We present a conceptual model of this study based on the hypotheses and research questions discussed so far (see Figure 1).

## 3. Methodology

### 3.1. Procedure and Participants

We tested our hypotheses and research questions using an online survey. The survey was conducted between 20 February and 26 February 2019. Survey participants were recruited, for an online survey, from online panel directory of a Korea Research Company. An email invitation for an online survey was sent to 6353 potential respondents, and out of 958 who actually participated in the survey, 510 respondents completed the survey. The mean age of participants was 41.1 (*SD* = 12.6 years), and 49% of the participants were female.

### 3.2. Measurement

#### 3.2.1. SNS Dependency (Learning, Entertainment)

SNS dependency was measured using the items used in previous studies [20,47]. Specifically, respondents were first asked about the SNS service they use most often in their daily lives and then answered how useful they think the SNS (they chose) has been in their everyday lives in satisfying the goals, on a five-point scale ranging from 1 (not helpful at all) to 5 (very helpful). SNS learning dependency was measured by asking respondents’ reliance on SNS for orientation and understanding. Specifically, the questions asked how helpful the SNS has been in their everyday lives to achieve the goal of “action orientation,” “interaction orientation,” “self understanding,” and “social understanding.” Likewise, SNS entertainment dependency was measured by asking the extent to which respondents think the SNS has been helpful to achieve the goal of “solitary play” and “social play.” A composite variable was created by using the mean score of the total item values for SNS learning dependency (Cronbach’s α = 0.88, *M* = 3.09, *SD* = 0.68) and for SNS entertainment dependency (Cronbach’s α = 0.80, *M* = 3.26, *SD* = 0.76).

#### 3.2.2. Policy Support

Policy support was measured by asking respondents six questions. The questions included if respondents support making provisions for the fine dust issue, penalizing companies that emit pollutants that cause the fine dust pollution, prosecuting business shutdown on companies that do not adhere to guidelines, providing incentives to companies that install facilities designed to minimize fine dust, establishment of a research fund for reducing fine dust problem, and collaborating with foreign countries to mitigate the fine dust pollution. Responses were measured on a 5-point scale, ranging from 1 (not at all) to 5 (strongly support), and based on the mean score for each item, a policy support index was created (Cronbach’s α = 0.88, *M* = 4.03, *SD* = 0.62).

#### 3.2.3. General and Personal Risk Perceptions

General risk perception was measured by asking respondents how threatening they think the fine dust problem is to people in general, using 7-point scale ranging from 1 (not at all) to 7 (extremely) (*M* = 5.82, *SD* = 1.13). Likewise, personal risk perception was assessed with a question asking how threatening they think the fine dust problem is to themselves, again using 7-point scale ranging from 1 (not at all) to 7 (extremely) (*M* = 5.95, *SD* = 1.12).

#### 3.2.4. Control Variables

To control for other variables that can affect the main variables in the current study, socio-demographic, as well as general media use, variables were included in the analyses. Specifically, socio-demographic factors, such as age (*M* = 41.1, *SD* = 12.6), gender (male = 51.2%), educational attainment (high school or less: 24.9%; college or bachelor’s degree: 64.5%; more than bachelor’s degree: 10.6%), and political orientation (ranged from 1–5, higher score indicating more conservative; *M* = 3.65, *SD* = 1.12) were included as control variables in the analyses. In addition, traditional media (e.g., television, radio, newspaper) (ranged from 1–5, with a higher score indicating more time spent on the media; *M* = 2.65, *SD* = 1.50) and general SNS use (ranged from 1–5, with a higher score indicating more time spent on SNS; *M* = 3.23, *SD* = 2.31) were used as control variables.

### 3.3. Analysis Strategy

First, we conducted correlational analyses between the variables of interest for preliminary analyses purposes (Table 1). To investigate the relationships among SNS dependency, perceptions of general and personal risk, and policy support, we ran a path analysis in STATA 14.2 using Structural Equation Modeling. As discussed earlier, factors such as age, gender, education, political orientation, as well as media use variables, were included as control variables in SEM analyses. In addition, to confirm the mediation relationships suggested in the path analysis, independent tests of significance of indirect effects were also conducted, employing a series of bootstrapping and bias-corrected confidence intervals analyses [55].

## 4. Results

### 4.1. Direct Influences

As shown in Table 1, the correlational analyses indicate that age (β = 0.18, SE = 0.02, *p* < 0.001), female (β = 0.10, SE = 0.04, *p* < 0.05), education (β = 0.12, SE = 0.03, *p* < 0.01), and traditional media use (β = 0.23, SE = 0.02, *p* < 0.001) were positively associated with policy support, whereas political conservatism was negatively associated with support for policy (β = −0.15, SE = 0.02, *p* < 0.001). The negative relationship between political conservatism and policy support seems to make sense considering the fact that the current ruling party of South Korea is the Democratic Party. The level of education (β = 0.12, SE = 0.03, *p* < 0.01) and traditional media use (β = 0.10, SE = 0.02, *p* < 0.05) were positively associated with perceptions of personal risk. General SNS use was associated with neither general risk perception (β = 0.09, SE = 0.02, *p* = 0.14) nor policy support (β = 0.09, SE = 0.01, *p* = 0.12).

**Figure 2:**
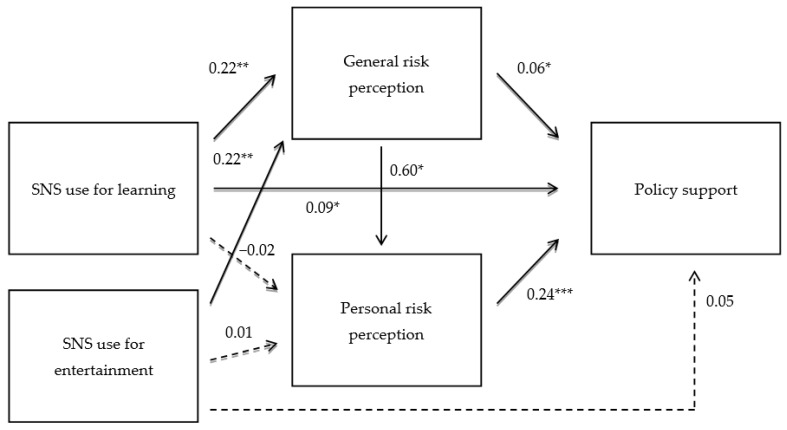
Results of hypothesized model of the study. Note. All the coefficients are standardized. Dotted lines represent insignificant results. * *p* < 0.05, ** *p* < 0.01, *** *p* < 0.001.

The proposed structural model produced the following indices, suggesting a good fit with the data: χ^2^/df = 1.50; *p* = 0.130; CFI = 0.990, TLI = 0.980, RMSEA = 0.031 (Figure 2). H1 was to examine the relationship between SNS learning dependency and perceptions of risk to people in general. The results showed that SNS learning dependency is positively associated with the perception that the risk is a serious threat to people (β = 0.22, SE = 0.08, *p* < 0.01), supporting H1. However, SNS learning dependency was not significantly related to the perception that the risk poses a threat to oneself (β = −0.02, SE = 0.06, *p* = 0.70) (RQ1). Regarding Q2, SNS entertainment dependency was associated with general risk perception (β = 0.22, SE = 0.07, *p* < 0.01) but not with personal risk perception (β = 0.01, SE = 0.05, *p* = 0.77). H2 was to assess the relationship between SNS learning dependency and policy support. The results indicated that SNS learning dependency is positively associated with policy support (β = 0.09, SE = 0.03, *p* < 0.01), lending support for H2. As expected, SNS entertainment dependency was not associated with policy support (β = 0.05, SE = 0.03, *p* = 0.12). In addition, general risk perception (β = 0.06, SE = 0.03, *p* < 0.05) and personal risk perception (β = 0.24, SE = 0.03, *p* < 0.001) were positively linked to policy support, supporting H3 and H4. The perception of general risk was positively linked to the perception of personal risk (β = 0.60, SE = 0.04, *p* < 0.001), confirming H5.

### 4.2. Indirect Influences

To confirm the mediating effects of perceptions of risk suggested in the path analyses, we conducted independent tests of significance of indirect effects, employing a series of bootstrapping and bias-corrected confidence interval analyses [55]. As shown in Table 2, the results showed that SNS learning dependency increased policy support through the perception of general risk (β = 0.05, *SE* = 0.02, 95% CI: [0.014, 0.078]), lending support for H6. We also examined whether there is a serial mediation relationship between SNS learning dependency, perceptions of general and personal risk, and policy support. The results confirmed that SNS learning dependency increases support for policies to mitigate the risk through the perception of general risk and, subsequently, the perception of personal risk (*b* = 0.04, *SE* = 0.01, 95% CI: [0.012, 0.064]), providing support for H7.

## 5. Discussion

### 5.1. Main Findings

The primary goal of this study was to examine how SNS dependency is associated with policy support to mitigate a risk, focusing on the role of two distinct types of risk perceptions—general and personal risk perceptions—in the process. First, the findings of this study indicate that SNS learning dependency was positively associated with general risk perceptions. This suggests that, when individuals rely on SNS for obtaining information and learning about people and society, this is likely to lead them to recognize the potential threat of a risk to the public. This finding is in line with previous research that showed SNS use was significantly related to increased societal-level risk perceptions [41].

The findings also show that neither SNS learning dependency nor SNS entertainment dependency were associated with the perception of personal risk. On the one hand, this finding demonstrates the strong impact of the psychological barrier where people believe that they are safer and away from dangers [56]. Even when individuals rely on SNS for understanding, the implications of events and issues around them and obtaining information, their psychological bias might prevent them from regarding the risk as relevant to themselves. Indeed, as the impersonal impact hypothesis [40] suggests, the influence of media on personal risk perception seems to be significantly lessened by impersonal impact—even in the case of SNS. On the other hand, the finding that SNS dependency was not associated with personal risk perception could be due to the measurement of SNS impact. For example, while Wu and Li [18] found a positive relationship between the use of SNS and perceptions of personal risk, they examined SNS involvement by asking participants the SNS activities they conducted *related to the environmental risk*. That is, they measured how participants’ specific use of SNS (i.e., writing, posting about the risk issue) was associated with risk perceptions. Since the present study examined how general SNS dependency (learning, entertainment) was linked to risk perceptions, these different measures of SNS use might have caused the different findings.

One interesting finding of this study was that SNS entertainment dependency was also positively associated with general risk perceptions. That is, even when individuals rely on SNS for entertainment purposes, it still increased their perception that the risk may pose a serious threat to people in general. While this finding seems puzzling at first, the unique nature of SNS communication might provide explanations for this finding. As discussed earlier, SNS, as a viable medium for interpersonal communication, allows individuals to interact with other people freely and efficiently. Thus, regardless of the actual purpose of using SNS, this interaction process, itself, might have increased the sense of belonging and closeness to people in the community, boosting empathic perceptions for other people and society [57].

The findings of this study show that SNS learning dependency is positively related to the support for policies addressing the risk. Consistent with previous research demonstrating that SNS dependency facilitates political or civic behaviors [20], participants who relied more on SNS for learning expressed greater intention to support policies to reduce the fine dust pollution. Considering the literature suggesting that people are more likely to engage in collective actions when they see other people react to the problem [52], people who witnessed how other people think and feel about the given risk on SNS might have been motivated to engage in preventive actions as they comply with the pressure. In addition, as people had more chances to learn and understand about the problematic nature of the problem on SNS, this knowledge and understanding might have naturally increased their support for policies aimed at reducing the potential threat. Indeed, previous research showed that individuals who use SNS for understanding purpose experience a high level of closeness and intimacy with other people on SNS [57]. Furthermore, the emotional bonds that people formed with other members of the society might also have aided the process. While sharing personal experiences and feelings on SNS [3], people could have created increased empathic concern for others, thereby being motivated to eliminate the potential threat to other people and their communities.

It is also worthwhile to note that the perception of general risk was positively associated with the perception of personal risk. As the literature on communication and social psychology suggests that individuals’ beliefs about how others think and feel is critically associated with their own thoughts and feelings [58], individuals who believed that the issue might raise a risk to other people also believed that the issue might affect themselves. The significant relationship between general and personal risk perceptions shown in this study seems to underscore the fact that SNS, indeed, can be an effective platform for people to relate to problems other than their own. While people often do not have direct experiences of the risk, the indirect experiences from observing others’ thoughts and feelings on SNS may facilitate the perception that the risk can be a serious threat for themselves as well.

Another interesting finding of this study is that the perception of general risk mediated the impact of SNS learning dependency on policy support. Individuals’ perception that the fine dust pollution is a serious threat to people in general—not the perception that it is a serious threat to themselves—mediated the impact of SNS learning dependency on their intention to support measures that can be taken to mitigate the risk. While prior research shows that personal risk perception plays a greater role in changes in judgment and behavior than general risk perception, the findings of this study indicate that the perception of general risk can also play a crucial role in the effects of SNS. Indeed, while the impersonal impact hypothesis [40] suggests that the media has little to no impact on risk perception at the personal level and, consequently, has little impact on individuals’ judgment or behavior regarding the risks, this might not be the case when SNS use induces the perception of general risk. In our study, individuals with high SNS learning dependency were shown to believe that a risk can be serious to the public, and this perception of generalized risk to others induced their support for policies to reduce the potential threat to their society.

Perhaps more interestingly, our findings indicate a serial mediation process where SNS learning dependency increases general risk perception, which then increases personal risk perception and, then, the support for policies addressing the issue. Even though SNS dependency might not directly affect the perception of personal risk, it can still effectively boost engagement with the risk as it evokes the general risk perception, which then induces personal risk perception. Indeed, given that individuals’ tendency to underestimate their risk of experiencing risk-related negative events, relative to others, can be maladaptive in risk contexts as it demotivates people to engage in preventive actions [39], our finding indicates that SNS can be a space where individuals learn the relevance of risk to themselves and, thus, are motivated to participate in preventive behaviors.

### 5.2. Theoretical and Practical Implications

This study aimed to expand the current knowledge of the role of media and risk perceptions in public engagements with crises. Responding to the call of the literature that emphasizes the importance of spelling out the target of risk in examining the effects of media use [15,19], this study offers further empirical support for the distinctive role of general and personal level risk perceptions in the impact of SNS.

Our study offers insights into our understandings about how we can better examine and understand the impact of SNS in crisis contexts. Despite the fact that individuals use SNS for various reasons and purposes, little research has examined the implications of relying on SNS for specific goals. The result of our study confirms that the extent to which people rely on SNS for learning can significantly influence their risk perceptions and policy support, unveiling the powerful impact of SNS dependency. In fact, while previous research on mass media suggests that the exposure to mass media with prosocial content increases the accessibility of prosocial thoughts, empathy, and behavior [59], the present study suggests that, without the exposure to specific prosocial contents, SNS dependency can influence the intention to support policies that can address a public health crisis. In other words, without purposeful or active search for news and information, SNS can exert critical influence on the perceptions and judgement of important social issues. Indeed, the findings of this study point toward the potential of SNS for increasing civic engagement.

The results of this study also have important practical implications. Policy makers and public officials have long been interested in enhancing public engagement with important social issues, as policies aimed at mitigating public risk often fail due to people’s disinterest or inaction. The findings of the present study suggest that reliance on SNS for learning and understanding can, indeed, cultivate individuals’ empathic concerns for other people, thus promoting civic engagement in times of crises.

### 5.3. Limitations and Future Research

Despite the theoretical and practical importance of this study, it is important to note its limitations. First, while the findings indicate that SNS learning dependency increases perceptions of risk to people in general and policy support, it is not clear what factors or aspects of SNS contribute to these perceptions. While the focal interest was to examine how SNS dependency, as a whole, can instigate changes in judgments and behaviors, future research that examines the unique effect of specific contents will further illuminate the findings obtained from the present study.

Additionally, we examined individuals’ engagement with risk by measuring the extent to which they support policies addressing it. While this is a widely accepted way of determining the public engagement of social issues, future research could investigate whether our understanding of the effects of SNS dependency might change depending on the type of engagement used in the study. For instance, the impact of reliance on SNS may be shown differentially for a passive (e.g., signing a petition online) and an active form of engagement (e.g., attending a political protest). Relatedly, it would be interesting to examine whether the perceptions of general and personal risks have differential impacts based on such distinctions. For instance, previous work found that the policy attitude was driven by general risk rather than personal risk [19], implying that general risk perception may be more significantly related to public judgments. Thus, the relationship between different levels of risk perception and different forms of engagement warrants additional attention.

Further, the influence of SNS dependency on public or civic engagement may be moderated by other media channels or the type of interpersonal networks. For example, it is possible that individuals with greater or various interpersonal networks, by being aware of cases of people directly affected from the risk and empathize with them, are more likely to participate in activities to reduce the problem. In addition, given that this study was conducted in the context of an environmental health crisis, particularly in South Korea, it would also be advantageous to investigate how the effects of SNS dependency on engagement will be shown in other issue domains and in other cultures. While the focus of the present study was to examine the cognitive mechanism underlying the impact of SNS dependency, exploring the emotional mechanism could add further insights into how SNS shape important social judgment and behavior.

In addition, considering the findings of prior work revealing that the effects of media vary by the type of risk (e.g., man-made or natural disaster), future research could examine how the impact of SNS might change depending on the types of risk. Last but not least, future research could examine the alternative relationship between SNS dependency and perceptions of risk, considering previous work that found that perception of threat increased dependency on interpersonal communication [60]. Examining how perceived risk may also increase the level of media dependency will further elucidate the role of perceptions of risk in the impact of SNS. By taking these limitations into account, future research could continue seeking to unravel the motivational and psychological mechanism underlying the social impact of SNS dependency. These future endeavors will illustrate a clearer picture of how, and under what conditions, SNS can promote public engagement with important social issues in the context of crises.

## 6. Conclusions

This study explored how the use of SNS in the context of a public health crisis is related to two distinct types of risk perceptions—general and personal risk perceptions—and how this, in turn, affects engagement behavior. Expanding the concept of media system dependency from MSD theory [36], we showed that SNS dependency (learning, entertainment) is distinctively associated with perceptions of general and personal risk and the support for policies to mitigate the risk. In general, the findings of this study suggest that SNS can debunk the perception of self-invulnerability by influencing individuals’ perception that people are being affected by the risk and, hence, it can affect themselves as well, which would subsequently lead to prosocial and participatory behavior.

## Figures and Tables

**Table 1:** Pearson correlations among the variables of this study.

Variables	1	2	3	4	5	6	7	8	9	10
1. Age	—									
2. Gender	0.01	—								
3. Education	0.07	−0.10 *	—							
4. Partisanship	−0.02	-0.05	−0.09 *	—						
5. Traditional media use	0.22 ***	0.07	0.07	−0.01	—					
6. General SNS use	−0.07	0.11 *	0.01	−0.05	0.036 ***	—				
7. SNS learning dependency	0.10 *	0.00	−0.01	0.04	0.18 ***	0.12 **	—	—		
8. SNS entertainment dependency	−0.04	0.02	−0.05	0.06	0.11 *	0.13 **	0.76 ***			
9. General risk perception	0.02	0.03	0.05	−0.09 *	0.08	0.09	0.13 **	0.14 **	—	
10. Personal risk perception	0.03	0.03	0.12 **	−0.06	0.10 *	0.16 **	0.04	0.07	0.61 ***	
11. Policy support	0.18 ***	0.10 *	0.12 **	−0.15 ***	0.23 ***	0.09	0.15 ***	0.10	0.40 ***	0.57 ***

Note. * *p* < 0.05. ** *p* < 0.01. *** *p* < 0.001.

**Table 2:** Indirect effects of SNS learning dependency on policy support via general and personal risk perceptions.

Paths			Indirect Effects	95% Confidence Interval
SNS learning dependency →	GRP →	Policy support	0.05 (0.02)	[0.014, 0.078]
SNS learning dependency →	PRP →	Policy support	0.07 (0.07)	[−0.071, 0.216]
SNS learning dependency →	GRP → PRP →	Policy support	0.04 (0.01)	[0.012, 0.064]

Note. GRP = General risk perception, PRP = Personal risk perception. Standardized coefficients and standard errors are shown.

## Data Availability

Data are available on request due to ethical issues.
